# Steroid-sensitive nephrotic syndrome in children: triggers of relapse and evolving hypotheses on pathogenesis

**DOI:** 10.1186/s13052-015-0123-9

**Published:** 2015-03-21

**Authors:** Samuel N Uwaezuoke

**Affiliations:** Department of Paediatric Nephrology Unit, University of Nigeria Teaching Hospital, Postal code- 400001 Ituku-Ozalla, Enugu, Enugu State Nigeria

**Keywords:** Relapse, Steroid-sensitive nephrotic syndrome, Children, Pathogenesis

## Abstract

Nephrotic syndrome remains the most common manifestation of glomerular disease in childhood. Minimal change nephropathy is the most common cause of the syndrome in children. Despite its initial high response rate to corticosteroids and its favorable prognosis, relapses are common leading to increased morbidity and cost of treatment.

This review seeks to appraise the common triggers of relapse and to highlight the evolving hypotheses about the pathogenesis of the syndrome. Literature search was conducted through PubMed, Google web search and Cochrane Database of Systematic reviews using relevant search terms.

Acute respiratory infections and urinary tract infections are the most frequent infectious triggers of relapse. Targeted interventions like initiating corticosteroid or its dose-adjustment during episodes of acute respiratory infection and zinc supplementation are reportedly effective in reducing relapse rates. Hypotheses on pathogenesis of the syndrome have evolved from the concepts of ‘immune dysregulation’, ‘increased glomerular permeability’ to ‘podocytopathy’.

Although development of drugs which can regulate the pathways for podocyte injury offers future hope for effective and targeted treatment, the relapse-specific interventions currently contribute to significant reduction in disease morbidity.

## Introduction

Nephrotic syndrome refers to the tetrad of edema, ‘nephrotic- range’ proteinuria, hypoalbuminemia, and hyperlipidemia. Nephrotic- range proteinuria in the pediatric age group is defined as protein excretion of more than 40 mg/m^2^/hour. In children, 24 hour-urine collections are unreliable. Thus, a single, first morning urine sample is now preferably used to quantify protein excretion by the estimation of the urine protein/creatinine ratio [1]. From the new management guidelines released by the Kidney Disease: Improving Global Outcomes (KDIGO) group, urine protein/creatinine ratio of ≥ 2000 mg/g correlates with ‘nephrotic-range’ proteinuria or dip-stick proteinuria of 3+ [2].

Nephrotic syndrome remains the most common manifestation of glomerular disease in childhood [3]. The International Study of Kidney Disease in Children (ISKDC) noted in a report that the vast majority of pre-adolescent children with idiopathic nephrotic syndrome had minimal change nephropathy (MCN) on renal biopsy [4]. This histological subtype is the most common cause of nephrotic syndrome in children [5]. More than 90% of children with minimal change nephropathy achieve remission with oral corticosteroids and thus have steroid-sensitive nephrotic syndrome (SSNS) [6,7]. By contrast, the majority of children with the second most common histological subtype- focal segmental glomerulosclerosis (FSGS) - do not respond to corticosteroids. Only 20% respond to the medication with a high risk of developing end-stage renal failure [5,8].

Despite the initial response rate of 90% to 95% and the favorable prognosis in children with SSNS, relapses occur in 60% to 90% of the initial responders which can lead to increased morbidity, complications and decreased quality of life [7,9,10]. The disease progresses to frequent relapses, often accompanied by steroid dependence in about 20% to 60% of patients [11]. Relapses are also associated with the risk of complications such as sepsis, thrombosis, dyslipidemia and malnutrition [12], while treatment with high-dose prednisolone is associated with significant adverse effects like hip avascular necrosis, hypertension, diabetes and behavioral disorders [13]. These challenges in management have led to the use of various long-term immunosuppressive and steroid-sparing strategies to reduce the frequency of relapses which include long-term, low-dose alternate day prednisolone, cyclophosphamide, levamisole, calcineurin inhibitors such as cyclosporine A (CsA) or tacrolimus, mycophenolate mofetil (MMF) and rituximab.

It is currently recognized that at least 50% of relapses are triggered by a viral upper respiratory tract infection [14-16]; which may be linked to non-specific host response to infection (cytokine release) rather than to viral antigen or antibody response [15]. Thus, other infections such as urinary tract infection (UTI), diarrhea, peritonitis and skin infections have also been implicated [14]. Studies have established the role of UTI as an important cause of relapse, as well as the cause of poor response to steroid therapy [17-19]. Based on these reports which have identified specific triggers of relapse, targeted interventions have been shown to alter disease morbidity.

Interestingly, the recent KDIGO Summary of Recommendation statements in the clinical practice Guideline for Glomerulonephritis advises “that daily prednisolone be given during episodes of upper respiratory tract and other infections to reduce the risk for relapse in children with frequently relapsing and steroid dependent SSNS already on alternate day prednisolone” [20].

This review seeks to appraise the triggers of relapse, as well as to highlight the evolving hypotheses about the pathogenesis of nephrotic syndrome.

### Literature search: strategy and outcome

Literature search was conducted through PubMed, Google web search and Cochrane Database of Systematic reviews over a period of six months (July to December, 2014). Using a combination of the following search terms ‘nephrotic syndrome in children’, ‘pathogenesis’, ‘triggers of relapse’, ‘zinc supplementation’, ‘steroid use in preventing relapse’ and ‘immunosuppressive drugs’, the search yielded 6,060 results by Google, 145 results by PubMed and 11 Cochrane reviews. Prospective and retrospective studies relevant to the objectives of the review and published in peer-reviewed journals were subsequently selected. Information was also gathered from textbooks published within the past ten years.

### Pathogenesis of nephrotic syndrome: evolving hypotheses

The pathogenesis of nephrotic syndrome remains incompletely resolved despite the strong evidence of immune dysregulation, mainly involving cell-mediated immunity. The tendency of the disease to manifest and relapse after viral infections or an atopic episode, the association with HLA antigens and Hodgkin’s lymphoma, and the therapeutic response to steroids and cyclosporine support the ‘immune dysregulation’ hypothesis [21]. In fact, abnormalities of T cell subsets and/or function have been reported in a number of patients with minimal change nephropathy [22-24].

Antigen presentation to T lymphocytes leads to a polarized immune response namely type 1(dominated by interleukin 2) and type 2 (dominated by interleukins 4, 10 and 13). Type 1 cytokines predominate in cell-mediated immunity and type 2 cytokines in atopy and class-switching of B-cells for production of IgG4 and IgE [25]. The findings of increased plasma levels of IgE, relatively normal IgG4 and association with atopy suggest type 2 cytokine bias in patients with minimal change nephropathy [21]. Increased systemic production of representative cytokines, chiefly interleukin-4 is also reported while in vitro studies suggest that podocytes express receptors for interleukin-4 and interleukin-13; activation of these receptors by the respective cytokines might disrupt glomerular permeability resulting in proteinuria [26].

Another hypothesis that evolved was the role of a systemic circulating factor which might result in increased glomerular permeability in patients with minimal change nephropathy and focal segmental glomerulosclerosis [21]. Various vascular permeability factors have been implicated including vascular endothelial growth factor, heparanase and hemopexin [27]. Heparanase is thought to increase the permeability of glomerular capillary wall by breaking down heparin sulphate glucosaminoglycans. This degradation has long been postulated as a cause of increased glomerular permeability to proteins [28].

However, recent evidence suggests that the primary defect in idiopathic nephrotic syndrome might be at the level of podocytes (the glomerular visceral epithelial cell). Conventional immunosuppressive agents like glucocorticoids and CsA are now known to directly affect podocyte structure and function; challenging the “immune theory” of the pathogenesis of childhood nephrotic syndrome and portraying the disease as a ‘podocytopathy’ [29]. Injury to the podocyte can indeed occur in many immunological and non-immunological diseases of the kidney. Acquired podocytopathies like idiopathic MCN and FSGS are considered as immunological diseases.

A hypothesis to unify these concepts of immune dysregulation, increased glomerular permeability and podocytopathy is yet to be proposed [21], but the speculation that critical podocyte proteins are probably potential targets for T cell cytokines or vascular permeability factors still needs further confirmation [24,27].

Nevertheless, the podocyte evidently plays a key role both in the maintenance of the glomerular filtration barrier and structural integrity, as its injury and loss contribute to proteinuria and progressive sclerosis [30]. Immunosuppressive agents such as corticosteroids and calcineurin inhibitors (CsA and tacrolimus) are known to have direct effects on podocytes through regulation of some cytokines and several signaling pathways relevant for stabilizing their actin cytoskeleton, cell maturation and survival [30]. These non-immunological actions thus protect the podocyte against injury and loss resulting in their anti-proteinuric effect in nephrotic syndrome.

Although the pathogenic mechanism for nephrotic syndrome has traditionally focused on dysregulation of T cells, there is increasing evidence that B cells also play a role given the efficacy of rituximab in treating the disorder [31]. Rituximab is a chimeric monoclonal antibody against the CD 20 receptors on B cells initially used for depletion of B cells in a variety of neoplastic and immune-mediated disorders [32], but was first reported to treat a child with nephrotic syndrome in 2004 [33]. In steroid-dependent or resistant cases, partial or complete remission rates of 70-85% are documented with many patients able to stop their immunosuppressive medications [31]. The exact mechanism of action remains unclear but it has been proposed that induction of regulatory T cells may lead to a late effect on decreasing proteinuria long after completion of therapy [34]. Current evidence from observational studies suggests that rituximab can induce the remission of nephrotic syndrome in patients with membranous nephropathy [35-37], MCN [33,38,39], and FSGS [40,41].

### Relapse: common triggers and targeted interventions

The common patterns of relapse in SSNS include infrequent relapses (1 relapse in 6 months or 1–3 relapses in 12 months) [2]; frequent relapses (2 or more relapses in 6 months of initial response or 4 or more relapses in any 12 month-period); and steroid dependence (2 consecutive relapses during steroid therapy or within 2 weeks of its cessation) [2,42,43]. About 50%-60% of children with SSNS have frequent relapses or steroid dependence. Factors documented as predictors of frequent relapses include age younger 3 years at onset, delayed time to remission (after 7–9 days), occurrence of an early relapse (in the first 6 months after initial treatment) and short initial therapy [43-49].

Identification of triggers of relapse in SSNS has led to targeted interventions which aim to ameliorate disease morbidity. An appraisal of studies on the subject shows the prime role of infections in causing relapse [14-19] although one of the studies has also reported school events and up-coming hospital visits as triggers [16]. Amongst the infectious triggers, respiratory tract infection consistently ranks as the most prominent and frequent factor irrespective of geographical setting [14-16,50] (Table 1). However, urinary tract infection assumes a more frequent role in other studies [17-19].

**Table 1:** **Comparison of study findings on common triggers of relapse in SSNS in children**

**Country**	**Triggers**	**Frequency (%)**	**Study**
-Pakistan (Asia)	-Infections	−62.9%	Moorani KN [14]
	(i) *ARI* ^a^	−54.5%	(retrospective study)
	(ii) *Diarrhea*	−22.3%	
	(iii) *UTI* ^b^	−8.2%	
	(iv) Others	−15.0%	
	-Poor compliance	−10.4%	
	-Unknown	−26.7%	
-Canada (N. America)	*-ARI (URI)*	−69.0%	McDonald N et al. [15]
	-*no ARI (URI)*	−31%	(prospective study)
-Japan (Asia)	-*ARI (URI)*	−52%	Takahashi S et al. [16]
	-*School events*	−18%	(retrospective study)
	-Others	−30%	
-India (Asia)	-*URI* ^*c*^	−92%	-Gulati A et al. (2011) [50]
	-*Gastroenteritis*	−6%	(prospective study)
	-*Fever without localized signs*	−2%	

^a^ARI-acute respiratory infections; ^b^UTI-urinary tract infections; ^c^URI-Upper respiratory infections.

In these studies [14-16], the average prevalence rate of respiratory tract infection as a trigger of relapse was approximately 66.9%. It is note-worthy that two of the studies were prospective [15,50], while the rest were retrospective [14,16] with their obvious limitations. It is therefore not surprising that the KDIGO recommendation of initiating daily prednisolone during episodes of upper respiratory infections is tagged with a low quality of evidence (grade C) [20].

Nevertheless, prospective interventional studies indicate that relapses are significantly reduced when the maintenance doses of corticosteroids are increased at the onset of viral upper respiratory infections [51,52], or when daily corticosteroids are given during onset of viral upper respiratory infections [50,53]. To underscore the importance of respiratory infections as triggers of relapse, zinc supplementation has also been found to reduce relapses in children with SSNS [54,55], based on the documented role of zinc in preventing these infections in children [56,57].

In the non-blind, randomized controlled trial conducted in a tertiary health-care setting by Gulati et al. [50], 100 children aged 1 to 16 years with recently diagnosed idiopathic frequently relapsing nephrotic syndrome were studied and were eligible for therapy with long-term, alternate-day prednisolone (0.5 mg-0.75 mg/kg) with or without levamisole (2 mg/kg). They were randomized to either receive their usual alternate-day prednisolone daily for 7 days during intercurrent infections (intervention group) or continue alternate-day prednisolone (control group). Sixty eight patients were treated with alternate-day prednisolone alone while 32 patients received alternate-day prednisolone and levamisole. At the 12-month follow-up, there were 44 relapses (31 infection-associated) in the intervention group as compared with 76 relapses (56 infection-associated) in the control group. Patients in the intervention group showed significantly lower infection-associated relapse rates and lower total relapse rates (Table 2). There was no increase in steroid toxicity: cushingoid features were seen in 4 patients (intervention group) and 5 patients (control group) while 2 patients developed cataract.

**Table 2:** **Relapse rates and cumulative prednisolone dosage at 12-month follow-up**

	**Intervention group**	**Control group**	**Rate**	**p**
	**n = 49**	**n = 46**	**Difference**	
Infection-associated	0.7 ± 0.3 (0.6,1.1)	1.4 ± 0.5 (1.2,1.9)	0.7 (0.3,1.1)	<0.01
Relapses (episodes/patient per year)^a^				
Total relapses (episodes/patient per year)^a^	0.9 ± 0.4 (0.7, 1.2)	1.8 ± 0.5 (1.4, 2.2)	0.9 (0.4, 1.4)	<0.0001
			Mean	
			Difference	
Cumulative prednisolone (mg/kg per year)^b^	120 ± 32 (105,131)	138 ± 22 (112,144)	16 (−26, 38)	0.3

^a^Relapse rates are the mean incidence density rates ± SD (95% confidence interval).

^b^The data are expressed as the means ± SD (95% confidence interval).

-*Adapted and reproduced with permission from Gulati et al. Clin J Am Soc Nephrol 6:63–69, January, 2011.*

In another prospective study by Mattoo and colleague [51], 36 children with steroid-dependent, relapsing nephrotic syndrome were placed on a maintenance alternate-day prednisolone therapy of 0.5 mg/kg. The patients were prospectively divided into two groups with comparable age and sex distribution. Group 1 patients were advised to take daily prednisolone for 5 days starting at the time of the onset of an upper respiratory infection. Those in group 2 remained on alternate-day prednisolone during episodes of upper respiratory infection. At the end of the 2 year follow-up period, the total number of relapses in group 1 was 40 with a mean of 2.2 ± 0.87 per patient as compared with 99 relapses with a mean of 5.5 ± 1.33 per patient in group 2.

Furthermore, Abeyagunawardena and co-worker [52] conducted a randomized, double-blind, placebo-controlled crossover trial of 40 sequential patients receiving low-dose prednisolone (<0.6 mg/kg) on alternate days as maintenance therapy. At the first sign of a presumed viral upper respiratory tract infection, the children were examined and randomly allocated to take medicine A or B containing either prednisolone or placebo in the first viral upper respiratory tract infection and vice versa in the second episode of the infection. If the criteria for diagnosis of a viral upper respiratory infection were met, the new drug was prescribed on daily basis for 1 week at the same dose as that of the prednisolone being taken by the patient on alternate-day basis. The relapse rate after viral upper respiratory infection was 48% in the placebo group and 18% in the prednisolone group. Although all the three studies had some methodological flaws, they were all randomized controlled trials with adequate baseline similarities between groups, and they all demonstrated that a 5 to7-day course of daily prednisolone as the intervention during infectious triggers of relapse significantly reduced the risk of relapse; making it an easy treatment option which would be cost-effective compared to treating multiple relapses.

Zinc supplementation is another interventional measure noted to reduce relapse rates in patients with SSNS; reported in two randomized controlled trials.

In the double-blind randomized study in India [54], 81 patients with SSNS, aged 1–16 years were stratified into frequent relapsers (n = 52) and infrequent relapsers (n = 29). They were randomized to receive 12-months of therapy with recommended dietary allowance of zinc (10 mg/day) (n = 40) or placebo (n = 41). Patients with frequent relapses also received long-term, alternate-day prednisolone. Subjects receiving zinc showed a 20% lower frequency of relapses with 44.7% of the patients having sustained remission compared to 27.5% in the placebo group. Patients with frequent relapses receiving zinc showed a 28% reduction in relapse rates and a significantly higher likelihood of sustained remission. Similarly, in another randomized-controlled trial recently conducted in Pakistan [55], two groups of 60 nephrotic children aged 2 to 15 years who received zinc supplements and placebo for 6 months were compared. Despite similar pre-study relapses and zinc levels in both groups, the post-study relapses in zinc group were lower (28%) compared to the placebo group (34.5%). In addition, the relapse rate reduction was 43% after zinc supplementation compared to 27% reduction in placebo group. With respect to adverse reactions, metallic taste was observed in 10% of cases.

## Conclusions

For pediatricians and pediatric nephrologists, there is a strong evidence to institute relapse-specific interventions in children with SSNS. Despite the generally good prognosis of the disease in children, the associated frequent relapses may lead to increased morbidity and potentially fatal outcomes. For more than 5 decades, corticosteroids have remained the mainstay of treatment for children with nephrotic syndrome especially those with MCN although their target cell or mechanism of action in nephrotic syndrome is not clearly understood [29]. Nevertheless, the recognition of the crucial role of podocyte injury in nephrotic syndrome has resulted in many new studies which have identified several important molecular pathways that can regulate podocyte injury; giving new evidence that indicates that the disease results from podocyte dysfunction [29]. Development of drugs which can affect these pathways holds prospects for targeted and effective treatments for nephrotic syndrome in future.

The new discoveries however do not preclude the need for the use of immunosuppressive agents like corticosteroids or CsA since these drugs also directly affect podocyte structure and function. Thus, the KDIGO recommendation of prescribing daily prednisolone during episodes of upper respiratory infections remains a relevant intervention aimed to reduce the risk of relapse. In tandem with this recommendation is the use of zinc supplements which reduces both the frequency of respiratory tract infections and relapse rates. Prompt search and treatment for UTI should not be overlooked by clinicians managing children with nephrotic syndrome. These relapse-specific interventions can reduce the morbidity associated with frequent relapses and ultimately improve the child’s quality of life.

## Abbreviations

CsA: Cyclosporine A
FSGS: Focal segmental glomerulosclerosis
HLA: Histocompatibility antigen
ISKDC: International Study of Kidney Disease in Children
KDIGO: Kidney Disease: Improving Global Outcomes
MCN: Minimal change nephropathy
MMF: Mycophenolate mofetil
SRNS: Steroid-resistant nephrotic syndrome
SSNS: Steroid-sensitive nephrotic syndrome
UTI: Urinary tract infection
